# Analyzing expression and phosphorylation of the EGF receptor in HNSCC

**DOI:** 10.1038/s41598-019-49885-5

**Published:** 2019-09-19

**Authors:** Malte Kriegs, Till Sebastian Clauditz, Konstantin Hoffer, Joanna Bartels, Sophia Buhs, Helwe Gerull, Henrike Barbara Zech, Lara Bußmann, Nina Struve, Thorsten Rieckmann, Cordula Petersen, Christian Stephan Betz, Kai Rothkamm, Peter Nollau, Adrian Münscher

**Affiliations:** 1 Laboratory of Radiobiology & Experimental Radiation Oncology, Hubertus Wald Tumorzentrum – University Cancer Center Hamburg, Hamburg, Germany; 2 Institute of Pathology, Hubertus Wald Tumorzentrum – University Cancer Center Hamburg, Hamburg, Germany; 3 Department of Otolaryngology and Head and Neck Surgery, Hubertus Wald Tumorzentrum – University Cancer Center Hamburg, Hamburg, Germany; 4 Research Institute Children’s Cancer Center and Department of Pediatric Hematology and Oncology, Hubertus Wald Tumorzentrum – University Cancer Center Hamburg, University Medical Center Hamburg-Eppendorf, Martinistrasse 52, 20246 Hamburg, Germany

**Keywords:** Head and neck cancer, Growth factor signalling, Phosphorylation, Predictive markers

## Abstract

Overexpression of the epidermal growth factor receptor (EGFR) in head and neck squamous cell carcinomas (HNSCC) is considered to cause increased EGFR activity, which adds to tumorigenicity and therapy resistance. Since it is still unclear, whether EGFR expression is indeed associated with increased activity in HNSCC, we analyzed the relationship between EGFR expression and auto-phosphorylation as a surrogate marker for activity. We used a tissue micro array, fresh frozen HNSCC tumor and corresponding normal tissue samples and a large panel of HNSCC cell lines. While we observed substantial overexpression only in approximately 20% of HNSCC, we also observed strong discrepancies between EGFR protein expression and auto-phosphorylation in HNSCC cell lines as well as in tumor specimens using Western blot and SH2-profiling; for the majority of HNSCC EGFR expression therefore seems not to be correlated with EGFR auto-phosphorylation. Blocking of EGFR activity by cetuximab and erlotinib points to increased EGFR activity in samples with increased basal auto-phosphorylation. However, we could also identify cells with low basal phosphorylation but relevant EGFR activity. In summary, our data demonstrate that EGFR expression and activity are not well correlated. Therefore EGFR positivity is no reliable surrogate marker for EGFR activity, arguing the need for alternative biomarkers or functional predictive tests.

## Introduction

The epidermal growth factor receptor (EGFR) is one of the most prominent oncogenes in head and neck squamous cell carcinoma (HNSCC). The EGFR belongs to the family of receptor tyrosine kinases and activation by its ligands leads to trans-auto-phosphorylation at numerous tyrosine residues. The binding of adapter proteins can initiate diverse downstream signaling pathways such as MAPK, AKT or STAT signaling. The EGFR is reported to be expressed or even over-expressed in most HNSCC, assuming that this will lead to increased basal EGFR activity^1^. Since EGFR signaling is involved in several cellular mechanisms leading to tumorigenicity and resistance towards radio- and chemotherapy several inhibitory strategies such as inhibitory antibodies or small molecule inhibitors have been developed^2^. Inhibition by the monoclonal antibody cetuximab has already been shown to improve radio- and chemotherapy^3,4^ and is approved for HNSCC treatment. Although there is a huge variation in EGFR expression described for HNSCC tumors and cell lines^5,6^, cetuximab approval is irrespective of the EGFR status of the tumors. It is therefore not surprising that for both regimens - cetuximab in combination with radiotherapy in the curative setting and cetuximab in combination with chemotherapy in the palliative treatment - a huge heterogeneity has been described in terms of treatment response, with some tumors responding very well while others failed to respond^3,4^. Alternative anti-EGFR regimens even completely failed to improve survival in treating unselected patient cohorts^7–9^, which underlines the urgent need to understand the consequences of increased EGFR expression and to develop reliable biomarkers. Due to the fact, that EGFR expression is easy to examine in tumor biopsies most studies have focused on EGFR expression as a potential biomarker. However, it has already become clear that EGFR expression is no sufficient prognostic or even predictive biomarker. This was comprehensively reviewed by Bossi *et al*.^10^. This failure of EGFR expression as a reliable biomarker might be based on a lack of correlation between EGFR expression and activity, which would be of special importance for anti-EGFR strategies intending to block EGFR activity. Consequently, analysis of EGFR activity rather than the detection of EGFR expression might lead to more effective biomarkers. Since kinase activity is challenging to examine, EGFR phosphorylation has been used as a surrogate marker so far. But, it is still not clear if EGFR expression is or is not well correlated with EGFR phosphorylation or activity in HNSCC.

Here we analyzed the relationship between EGFR expression and activity in HNSCC. To this end, we detected EGFR protein expression and auto-phosphorylation in numerous HNSCC tumors and cell lines using a tissue micro array, Western blot and SH2-profiling.

## Results and Discussion

### EGFR expression in HNSCC specimens

**Figure 1:**
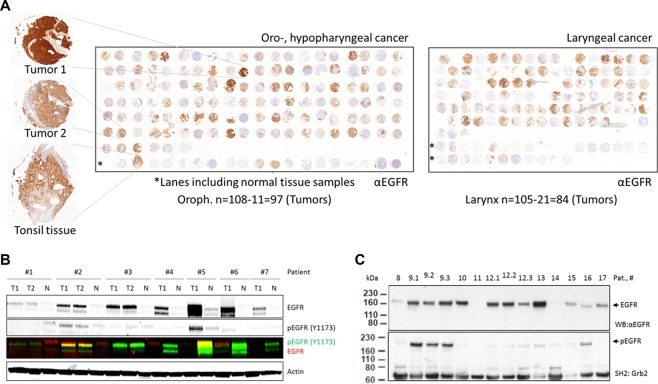
EGFR expression and phosphorylation in HNSCC tumor samples. (**A**) Immunohistochemical detection of EGFR on a tissue microarray of oro- & hypopharyngeal (left) and laryngeal (right) cancer samples. Nuclei were stained using hematoxylin. Rows marked with * show samples of different normal tissues. Far left: Exemplary tumor samples with strong and moderate EGFR expression and normal tonsil tissue with moderate expression. (**B**) EGFR expression and phosphorylation at Tyr1173 detected by Western blot in HNSCC tumor samples (T) and corresponding normal tissue samples (N). For three tumors two individual specimens were analyzed (T1 and T2). (**C**) EGFR expression and phosphorylation at Tyr1068, 1086 and 1148 in HNSCC tumor samples. EGFR expression was analyzed by Western blot and corresponding antibodies, EGFR phosphorylation was analyzed by SH2 profiling and far-Western blot using a Grb2-SH2 domain. For two tumors three individual specimens were analyzed (0.1–0.3). For B and C the same amount of protein was analyzed per sample. Furthermore, the blots were cropped after editing the intact images. The unedited and uncropped blots are shown in Fig. S1.

The aim of this study was to analyze the relationship between EGFR expression and activity/auto-phosphorylation in HNSCC. We first analyzed EGFR expression in HNSCC tumor samples. To enable comparability between the samples we used tissue micro arrays (TMA). The two previously described TMA include 224 samples from oropharyngeal, hypopharyngeal and laryngeal tumors^11,12^ (Fig. 1A) which were stained and analyzed under standardized conditions at the Institute of Pathology at the UKE. Although 85% of the samples display positive EGFR staining, we observed huge variations in EGFR expression with 28% of the tumors showing weak EGFR, 37% moderate and 20% very strong expression (Table 1). However, this was not correlated with tumor staging or grading (data not shown). A similar heterogeneity in EGFR expression has been described for HNSCC before^5,13^. Since normal epithelial tissue is inherently positive for EGFR expression, which is reflected by a moderate staining in some normal tissue samples, tumor-related upregulation of EGFR and therefore tumor-related EGFR overexpression might only be present in the subset of samples displaying strong staining^14^. Tumor-specific overexpression of EGFR is assumed to be caused by an amplification of the EGFR gene^15^ which can be found in 10–30% of HNSCC^5,15–17^. However, EGFR gene amplification and EGFR overexpression are not always correlated^5,17^. Furthermore, with respect to therapeutic strategies, which tend to inhibit EGFR activity, even overexpression of the EGFR protein might not reflect tumor specific hyperactivity. Receptor activity might better be addressed by analyzing EGFR auto-phosphorylation.

**Table 1:** EGFR scoring in HNSCC samples (TMA)

	negative	weak	moderate	strong	Sum
OroHypo	17	40	46	23	126
Lar	17	23	37	21	98
Sum	**34 (15%)**	**63 (28%)**	**83 (37%)**	**44 (20%)**	**224**

### EGFR auto-phosphorylation in HNSCC specimens

To analyze EGFR auto-phosphorylation (pEGFR) we chose snap-frozen tissue of tumor and corresponding normal tissue samples collected during surgical resection. We analyzed an independent cohort of seven HNSCC patients using Western blot. Again, we observed a heterogeneity ranging from weak to very strong signal intensities. Indeed, all tumor samples displayed increased EGFR expression compared to the corresponding normal tissue control. But surprisingly, EGFR expression was not correlated with EGFR auto-phosphorylation at Tyr 1173 (Fig. 1B). Furthermore, EGFR auto-phosphorylation was clearly upregulated only in two HNSCC samples (#2&#5) compared to the normal tissue. These results clearly show strong discrepancies between EGFR expression and auto-phosphorylation (activity) in patient samples.

So far auto-phosphorylation of EGFR was analyzed using an antibody directed against a single phosphorylated tyrosine residue (Tyr1173). Since EGFR signaling is regulated by the phosphorylation of multiple tyrosine and serine/threonine-residues^18^, we analyzed EGFR auto-phosphorylation on multiple tyrosine residues in a second cohort of 11 HNSCC patients by SH2-profiling using the GRB2 SH2-domain as a probe (snap-frozen tissue). Figure 1C shows once again a strong heterogeneity in EGFR expression and clear discrepancies between EGFR expression (upper panel) and EGFR auto-phosphorylation detected by the binding of the Grb2-SH2 domain known to recognize the phosphorylated tyrosine residues Tyr1068, Tyr1086 and Tyr1114, respectively^19^. Only one (#9) out of five samples which displayed strong EGFR expression also displayed strong EGFR auto-phosphorylation, while only one of four samples with low levels of EGFR expression also displayed weak EGFR auto-phosphorylation (#16). These strong discrepancies between EGFR expression and auto-phosphorylation are in line with data from Hama *et al*. showing no correlation between EGFR expression and auto-phosphorylation at Tyr1092in HNSCC samples^20^. Our results further indicate that intratumoral heterogeneity had only a limited influence under the chosen conditions since the same results were obtained for multiple individual pieces of the same tumor (Fig. 1B: for #2 & #3 two pieces each; Fig. 1C: for #9 & #12 three pieces each). Again, we did not detect any correlation between tumor staging or grading and EGFR phosphorylation.

### EGFR expression and auto-phosphorylation in HNSCC cell lines

**Figure 2:**
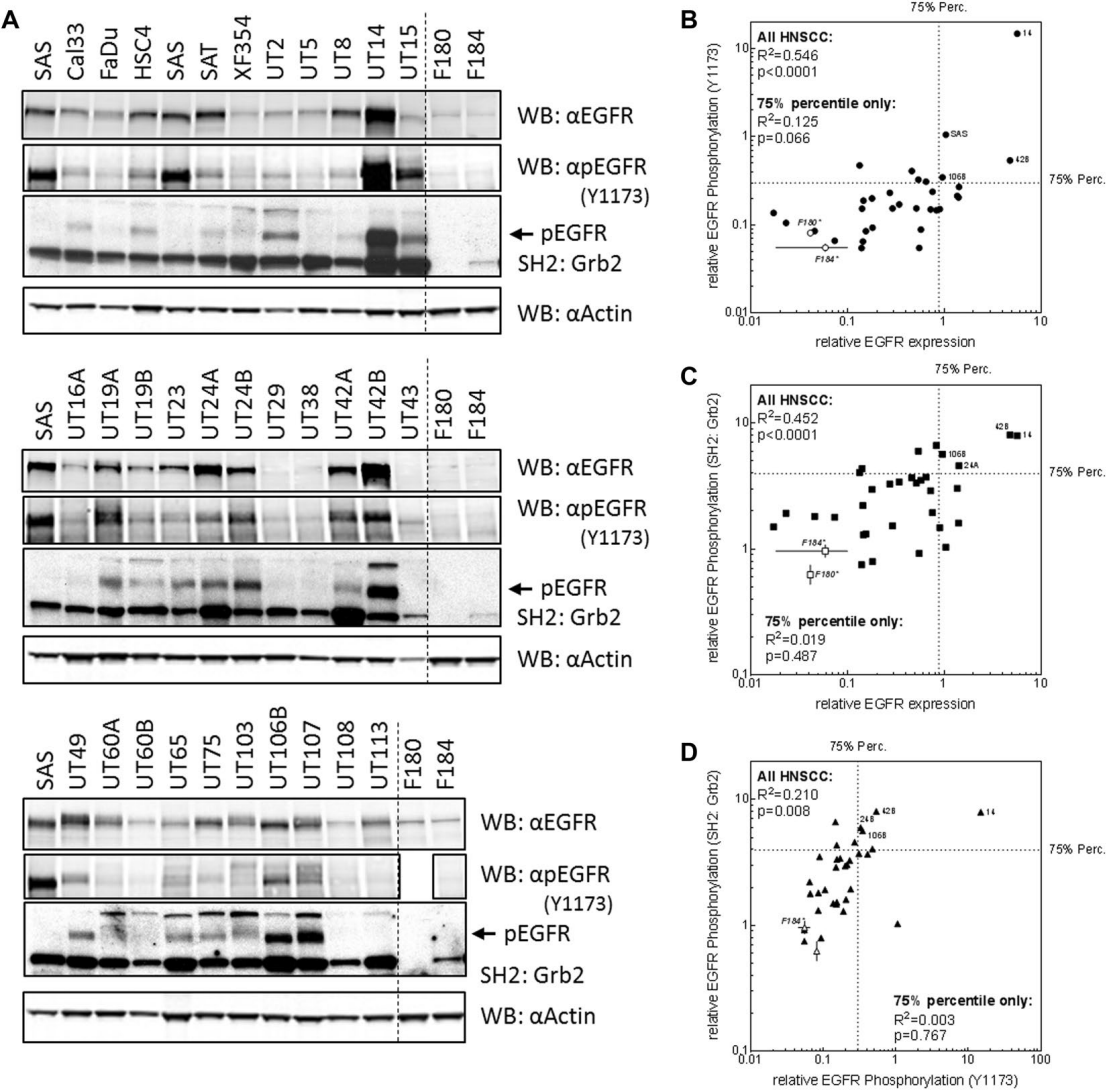
EGFR expression and phosphorylation in HNSCC cell lines. (**A**) EGFR expression and phosphorylation analyzed in 32 HNSCC cell lines by Western and far-Western blot using EGFR & pEGFR (Tyr 1173) specific antibodies and a SH2-domain from Grb2 (phosphorylation at Tyr1068, 1086 and 1148). An actin specific antibody served as control. SAS and normal fibroblasts F180 and F184 served as references. For each sample, the lysate of 40.000 cells was analyzed. Blots were cropped after editing the intact images. The unedited and uncropped blots are shown in Fig. S2. Signal intensities were plotted as indicated. (**B–D**) Correlation of (**B**) EGFR expression vs. phosphorylation at Tyr1173, (**C**) expression vs. phosphorylation at Tyr1068/ 1086/1148 and (**D**) phosphorylation at Tyr1173 vs. phosphorylation at Tyr1068/ 1086/1148.

Next we wanted to analyze EGFR expression and auto-phosphorylation in HNSCC under standardized conditions. To this end, we used a panel of 32 HNSCC cell lines and analyzed EGFR expression and Tyr1173 phosphorylation by Western blot while Tyr1068/1086/1114 phosphorylation was analyzed by SH2-profiling and far-Western blot (Fig. 2A). The same number of cells was loaded per lane, normal fibroblast cell lines (F180 & F184) served as controls and the lysate from SAS cells was used as an internal standard to enable comparability of the different blots. As in immunohistochemistry we observed a huge variation in EGFR expression with approximately 13% of the cell lines displaying actually no and 34% a weak signal. A moderate signal was observed for 31% of the cell lines while a clear overexpression (>75% percentile) could be observed in 22% of the cell lines. These percentages coincide very well with the percentages observed in the TMA. By detecting EGFR auto-phosphorylation either by Western blot or SH2-profiling we once again observed strong discrepancies between EGFR expression and auto-phosphorylation, comparable to those already observed for the tumor samples (Fig. 2A). Nevertheless, by correlating EGFR expression and auto-phosphorylation, significance was reached (p < 0.05) for both the detection of Tyr1173 and SH2-profiling (Fig. 1B,C). Although we analyzed a considerable number of cell lines, the correlation is only associated with a moderate determination coefficient (R^2^ > 0.4;). Since both correlations seem to be dominated by the overexpressing and hyper phosphorylated samples (<75% percentile) we reanalyzed the data excluding these samples in accordance to Kasten-Pisula *et al*.^6^. This lead to the loss of significance which demonstrates the low dependence of EGFR auto-phosphorylation level on EGFR expression level in the majority of HNSCC cell lines. Such independence of EGFR expression and auto-phosphorylation or activity would explain the failure of EGFR expression as a predictive marker for anti-EGFR therapy.

Some studies have demonstrated EGFR-phosphorylation to be a prognostic biomarker^20–23^. However, it has also been shown by Wheeler *et al*. that different sites might have different prognostic values (while Tyr1068 correlated with prognosis, Tyr992 did not)^21^, raising the question if EGFR-phosphorylation *per se* might be a reliable marker for EGFR-activity. With respect to the cell lines analyzed here, EGFR auto-phosphorylation at Tyr1173 was indeed positively associated with auto-phosphorylation at Tyr1068/1086/1114 as detected by SH2-profiling (Fig. 2D). But this correlation was also dependent on the samples with highly phosphorylated EGFR, demonstrating only a weak association of different EGFR phospho-sites.

### Inhibition of EGFR auto-phosphorylation

As demonstrated above, the phosphorylation of the EGFR at different sites is not strictly correlated, demonstrating that EGFR phosphorylation *per se* does not necessarily reflects EGFR kinase activity, which might help explain the results from Wheeler *et al*. discussed above. To test the correlation between EGFR phosphorylation and activity we inhibited EGFR by either cetuximab or erlotinib. We had previously analyzed the effect of EGFR inhibition by 30 nM cetuximab or 5 µM erlotinib on a single phosphorylation site (Tyr1173) in approximately half of the tested cell lines^24,25^. Due to the discrepancies observed for different phosphosites as described above (Fig. 2D), we now analyzed multiple phosphosites using SH2 domains from Grb2 (detecting autophosphorylation at Tyr1068/1086/1114) and from CRK and ABL2 for phosphotyrosine profiling after treatment with clinically relevant doses of cetuximab (30 nM) or erlotinib (5 µM). The latter ones recognize EGFR when phosphorylated at Y803/992/1101 and Y1148, respectively, as predicted by NetPhorest. We chose 8 HNSCC cell lines which differ in the amount of EGFR expression and phosphorylation. Five of them displayed weak (FaDu < UT-SCC 5 < SAS < UT-SCC 60A < Cal33) and 3 strong (UT-SCC 42A < UT-SCC 15 < UT-SCC 14) EGFR auto-phosphorylation as previously detected by SH2 profiling (see Fig. 2A). To demonstrate comparable loading between treated and untreated cells we also detected EGFR and ERK1/2 expression. However, please note that signals are not comparable between different cell lines because different gels and blots had to be used (see supplementary information, Fig. S3) and equal protein concentrations were loaded instead of equal cell numbers.

**Figure 3:**
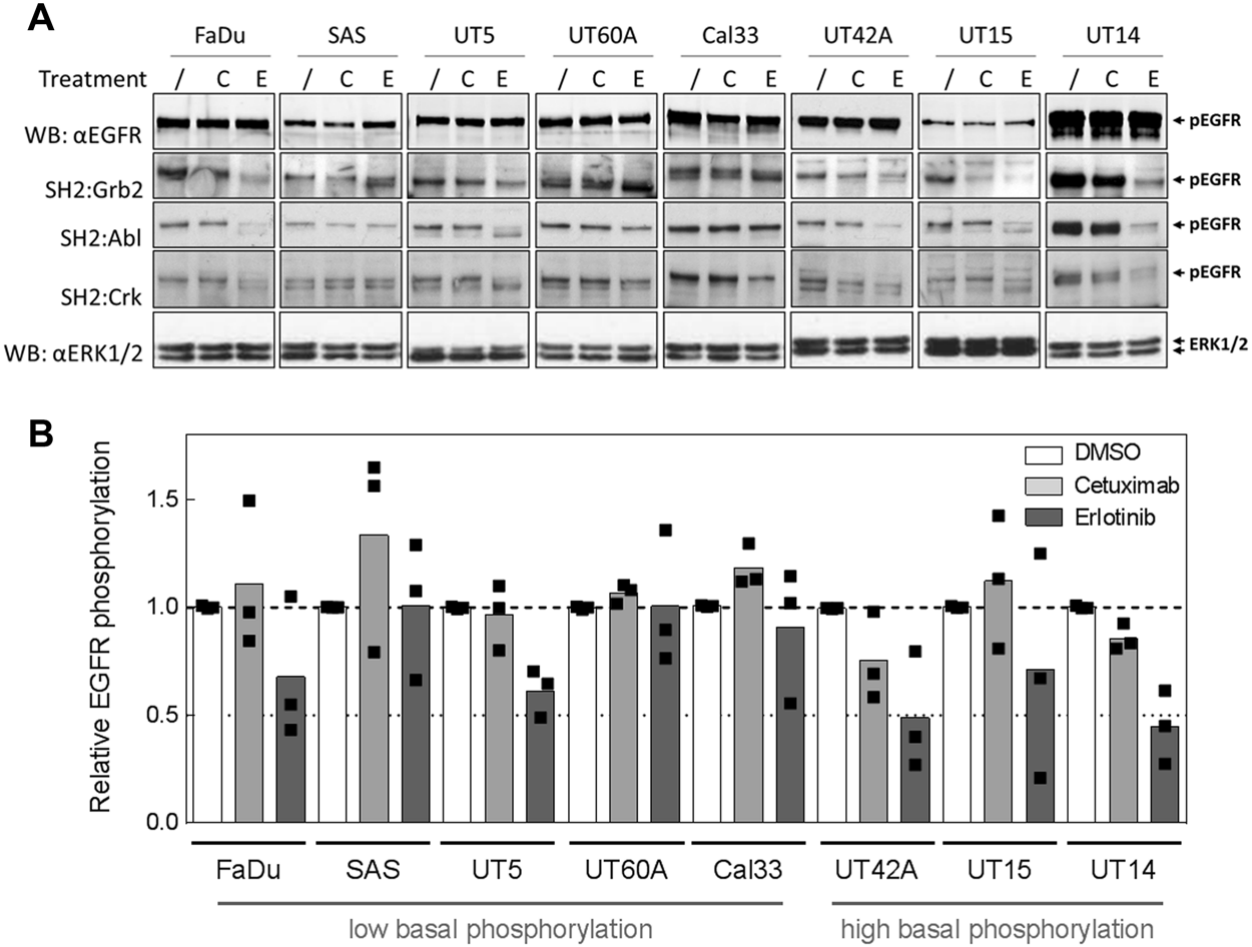
Inhibition of EGFR signaling. Eight HNSCC cell lines displaying either weak (FaDu < UT-SCC 5 < SAS < UT-SCC 60A < Cal33) or strong (UT-SCC 42A < UT-SC 15 < UT-SCC 14) EGFR phosphorylation as detected by SH2-profiling (see Fig. 2A) were treated with 30 nM cetuximab or 5 μM erlotinib for 2 h. (**A**) Cell lysates were analyzed either by Western blotting (αEGFR, αERK1/2) or using SH2-profiling and far-Western blot using SH2-domains from Grb2, Abl2 and CRK. Equal amounts of protein were loaded per lane and loading was controlled by ERK1/2 detection. Blots were cropped after editing the intact images. The unedited and uncropped blots are shown in Fig. S3. (**B**) Quantification. The pEGFR signals detected by SH2 profiling were normalized to the corresponding EGFR signals. For each cell line, signals were further normalized to the DMSO control.

EGFR inhibition again reveals discrepancies between the individual phosphosites: While cetuximab for example causes a reduced signal in UT-SCC 15 cells when using the Grb2-domain for detection, there was no reduction detectable when using the CRK- or the ABL2-SH2 domain (Fig. 3A). However, when pooling the data from all three SH2 domains, a clear inhibition after cetuximab could be observed only for UT-SCC 42A and UT-SCC 14. In all three cell lines with high basal phosphorylation a reduction in EGFR auto-phosphorylation was observed after erlotinib (Fig. 3A,B). Besides reduced pEGFR signal intensities we even observed a shift towards lower molecular weight, especially in UT-SCC 15 cells, also indicating reduced phosphorylation and therefore inhibited activity. Interestingly we observed such a shift and reduced EGFR phosphorylation after erlotinib treatment also in two of the cell lines with low basal phosphorylation (FaDu & UT-SCC 5), indicating inhibition of EGFR activity in these cells, too. The other three cell lines with low basal phosphorylation (SAS, UT-SCC 60A and Cal33) did not show a notable reduction of EGFR auto-phosphorylation.

Given the obvious discrepancies between the effects mediated by cetuximab and erlotinib it has to be stated that the effect of cetuximab on EGFR auto-phosphorylation also depends on other factors such as the K521 polymorphism in the EGFR gene^24^. In contrast, EGFR inhibition by erlotinib seems to be independent of such genetic alterations. The polymorphism confers resistance towards cetuximab on the level of Tyr1173 auto-phosphorylation and cell inactivation, which might be related to a slightly reduced binding affinity^24^. These findings fit quite well with the data presented here, since none of the K521 polymorphism positive cells (FaDu, SAS, UT-SCC 5 and UT-SCC 60A) show reduced EGFR auto-phosphorylation following cetuximab treatment, whereas erlotinib-treated FaDu and UT-SCC 5 cells show reduced EGFR auto-phosphorylation.

Taken together, our data demonstrate reduced auto-phosphorylation after EGFR inhibition especially in cells with high basal EGFR phosphorylation level. This points to substantial basal EGFR activity in these cells. Yet, cells with low basal EGFR phosphorylation may also harbor relevant EGFR activity, as demonstrated by reduced EGFR auto-phosphorylation in FaDu and UT-SCC 5 cells after erlotinib treatment. These findings are supported by the observation that erlotinib also causes a dramatic inhibition of UT-SCC 5 proliferation^25^. It is important to underline that EGFR expression levels do not seem to play a role in this context, since UT-SCC 60A cells, which have strong EGFR expression but low basal auto-phosphorylation, are quite resistant to EGFR inhibition. On the other hand, UT-SCC 15 cells, which express EGFR on a low level but have a high basal auto-phosphorylation, are more sensitive. Our data also highlight the different efficiencies if EGFR is inhibited either by small molecule inhibitors like erlotinib or antibodies like cetuximab. While cetuximab is sensitive towards alterations also in the extracellular domains of the EGFR, erlotinib is more efficient in blocking EGFR auto-phosphorylation. Of course, small molecule inhibitors such as erlotinib tend to be more unspecific compared to inhibitory antibodies. Nevertheless, reduced auto-phosphorylation after erlotinib treatment strongly indicates reduced EGFR activity.

Therefore, we can conclude that increased basal EGFR auto-phosphorylation represents notable EGFR activity. Relatively low EGFR auto-phosphorylation, on the other hand, does not necessarily indicate negligible EGFR activity and therefore does not rule out the chance to target a tumor via EGFR inhibition.

The most important result of this study is the lack of correlation between EGFR protein expression and EGFR phosphorylation. This can be explained by the fact, that EGFR phosphorylation and activity are regulated by multiple factors, such as the presence of EGFR-specific ligands or dimerization partners and the ability to shut down the EGFR-mediated signaling by either internalization or the activity of protein-tyrosine phosphatases^26^. Furthermore the EGFR is influenced by the expression/activity kinases such as Src^27^ or co-factors such as integrins^28^. Additionally, differences in the subcellular distribution, such as lower surface levels, can influence the EGFR activity. Although the latter explanation does not seem very likely for the analyzed HNSCC cell lines (since EGFR localizes predominantly on the surface of HNSCC cells^29^) we expect several of the mentioned factors to differentially influence EGFR phosphorylation in the individual cell lines and therefore to disrupt a possible correlation between EGFR expression and phosphorylation for the tested samples. Only if the EGFR is heavily overexpressed, a significant formation of spontaneous homodimers is likely to cause increased EGFR phosphorylation as observed for example for UT-SCC 14 or UT-SCC 42B cells.

## Summary

The results presented here clearly demonstrate large discrepancies between EGFR protein expression and auto-phosphorylation/activity in HNSCC cell lines as well as tumors. While the majority of HNSCC tumors displayed increased EGFR expression in comparison to the corresponding normal tissue, only a small number displayed increased EGFR auto-phosphorylation. For the HNSCC tumor samples as well as for most of the cell lines there was no correlation of EGFR expression and EGFR auto-phosphorylation. Inhibition of EGFR activity by cetuximab or erlotinib demonstrated relevant EGFR activity in cells with elevated basal EGFR auto-phosphorylation, which was independent of EGFR expression. In addition, however, also some cells with low basal EGFR auto-phosphorylation displayed relevant EGFR activity. Furthermore, EGFR signaling and the efficiency of EGFR inhibition also depend on additional factors such as EGFR polymorphisms and mutations in the corresponding downstream pathways^24,30^, which are not represented by the EGFR auto-phosphorylation status.

Based on our data EGFR expression is not a sufficient marker to determine EGFR activity. Although challenging to assess, basal EGFR auto-phosphorylation is a reasonable, but not conclusive, indicator for EGFR activity. Since this is of crucial importance for therapeutic anti-EGFR strategies and for understanding how EGFR-mediated processes contribute to tumorigenicity or therapy resistance, more effort is required to directly analyze the activity of EGFR or EGFR-dependent pathways in HNSCC and to establish functional assays for treatment prediction in order to enable an optimal personalized use of targeted therapeutics.

## Materials and Methods

### Tissue micro array (TMA)

The Hamburg TMA used in this study was described earlier in detail^11,12^. In brief, one 0.6 mm core per cancer was taken from formalin fixed, paraffin embedded tissue blocks of carcinomas from the oro-and hypopharynx (n = 126), and the larynx (n = 105). The usage of archived diagnostic left-over tissues for manufacturing of tissue microarrays and their analysis for research purposes as well as patient data analysis has been approved by local laws (HmbKHG, §12,1) and by the local ethics committee (Ethics commission Hamburg, WF-049/09). All work was carried out in compliance with the Helsinki Declaration and in accordance with the ethical guidelines and regulations of the University Medical Center Hamburg-Eppendorf (UKE). The samples were collected from the archives of the pathology department of the UKE. Immunohistochemistry (IHC) was performed on freshly cut TMA sections in a single experiment. EGFR immunostaining was performed using a monoclonal antibody (DAKO; clone: E30, dilution 1:50) using a DAKO autostainer Link48. Only membranous staining was evaluated. The staining intensity (0, 1+, 2+, 3+) was recorded for each tissue spot by an unbiased pathologist.

### Snap frozen tissue

Tumor and normal tissue from HNSCC patients was collected during surgical resection and was snap frozen and stored in liquid nitrogen. Tissue status (tumor/normal) was confirmed by an experienced pathologist after cryosectioning and H&E-staining (data not shown). The samples were collected and processed in accordance with UKE ethical guidelines and regulations, in accordance with the ethical standards of the institutional and/or national research committee, and with the 1964 Helsinki declaration and its later amendments or comparable ethical standards. All patients gave written informed consent. Furthermore, the ENT department has a biobank, which was notified to the Hamburg Representative for Data Protection and Freedom of Information (HmbBfDI) in accordance with §12 a para. 5 HmbKHG.

### Cell lines

HPV-negative HNSCC cells were grown in D-MEM medium (Invitrogen) containing 10% FCS (PAN Biotech) and 2 mM glutamine (Invitrogen) at 37 °C and 100% humidification. Cells were identified by a short tandem repeat multiplex assay (Applied Biosystems) if a reference was available. Cell lines UT-SCC-8, UT-SCC-14 and SAT harbor EGFR gene amplifications^6^.

### Western blotting

Proteins from whole cell extracts were detected by Western blot according to standard protocols. Primary antibodies: anti-EGFR (#4407; 1:1,000), anti-pEGFR (Y1173, #2239; 1:1,000) from Cell Signaling Technology; anti-actin (#A-2228; 1:40,000) from Sigma-Aldrich. Secondary antibodies: anti-mouse and anti-rabbit antibodies from LI-COR Biosciences (1:7,700). SeeBlue Plus2 Pre-stained Protein Standard (Thermo Fischer Scientific) or Novex Sharp Prestained Protein Ladder (Invitrogen) were used as protein standards.

### SH2 Profiling

Protein extraction for SH2-profiling was performed as previously described^31^. In brief, whole cellular protein extracts were separated by SDS-PAGE, proteins were transferred to PVDF-membranes (Thermo Fischer Scientific) blocked with 10% skim milk in TBST-buffer and incubated with bacterially expressed SH2 domains pre-complexed by streptavidin horseradish-peroxidase at a concentration of 1 µg/ml. Signals were detected by chemiluminescence (Pierce ECL Western Blotting Substrate from Thermo Fischer Scientific). SeeBlue Plus2 Pre-stained Protein Standard (Thermo Fischer Scientific) or Novex Sharp Prestained Protein Ladder (Invitrogen) were used as protein standards.

### Signal detection and quantification

Signals from Western blotting and SH2 profiling were either detected by chemiluminescence and X-ray films (Thermo Fischer Scientific) or fluorescence and Odyssey® CLx Infrared Imaging System (LI-COR Biosciences). Signal quantification was either performed by ImageJ or the Odyssey® CLx Infrared Imaging System (LI-COR Biosciences). If necessary, lysates of SAS cells was used for standardization.

## Supplementary information

Supplementary Dataset

## Data Availability

All data generated or analyzed during this study are included in this published article.
