# Supplementary material for: Analyzing expression and phosphorylation of the EGF receptor in HNSCC
Source: Sci Rep. 2019 Sep 19;9:13564. doi: 10.1038/s41598-019-49885-5 (PMC6753061; doi:10.1038/s41598-019-49885-5)

**Supplementary Information for:**

**Analyzing expression and phosphorylation of the EGF receptor in HNSCC**

Malte Kriegs^1#*^, Till Sebastian Clauditz^2#^, Konstantin Hoffer^1^, Joanna Bartels^3^, Sophia Buhs^4^, Helwe Gerull^4^, Henrike Barbara Zech^3^, Lara Bußmann^3^, Nina Struve^1^, Thorsten Rieckmann^1,3^, Cordula Petersen^1^, Christian Stephan Betz^3^, Kai Rothkamm^1^, Peter Nollau^4+^, Adrian Münscher^3+^

^1^Laboratory of Radiobiology & Experimental Radiation Oncology, Hubertus Wald Tumorzentrum – University Cancer Center Hamburg; ^2^Institute of Pathology; ^3^Department of Otorhinolaryngology and Head and Neck Surgery, Hubertus Wald Tumorzentrum – University Cancer Center Hamburg; ^4^Research Institute Children’s Cancer Center and Department of Pediatric Hematology and Oncology, Hubertus Wald Tumorzentrum – University Cancer Center Hamburg, University Medical Center Hamburg-Eppendorf, Martinistrasse 52, 20246 Hamburg, Germany

(^#^M.K. and T.S.C. share equal authorship; ^+^A.M. and P.N. share equal authorship)

*Corresponding author

Dr. Malte Kriegs

Laboratory of Radiobiology & Experimental Radiation Oncology

Hubertus Wald Tumorzentrum – University Cancer Center Hamburg

University Medical Center Hamburg-Eppendorf

Martinistr. 52

D-20246 Hamburg

Tel.: [+49] 40 7410 53594

Fax: [+49] 40 7410 55139

E-mail: m.kriegs@uke.de

**Fig. S1 Uncropped Western and far-Western (SH2 profiling) blots from Fig. 1B & C. A** Western blots depicted in Fig. 1B detecting EGFR (red) and pEGFR (green) on the same blot using fluorescent antibodies. Thereafter actin (green) was detected as well. The individual signals were depicted in Fig. 1B also in monochrome to improve clarity. **B** Western blot detecting EGFR and far-Western blots detecting pEGFR which are depicted in Fig. 1C. For detection chemiluminescence was used. The first lanes included samples from an unrelated experiment and were therefore obscured.

A


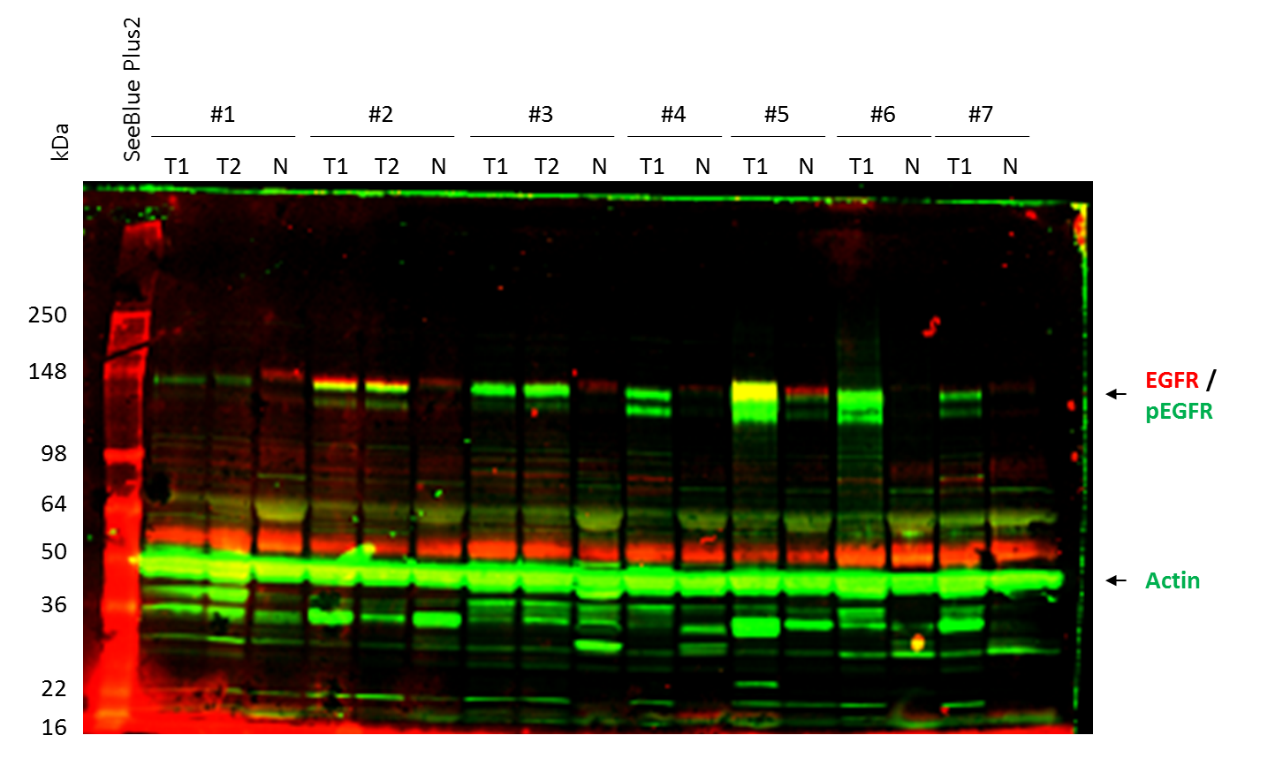


B


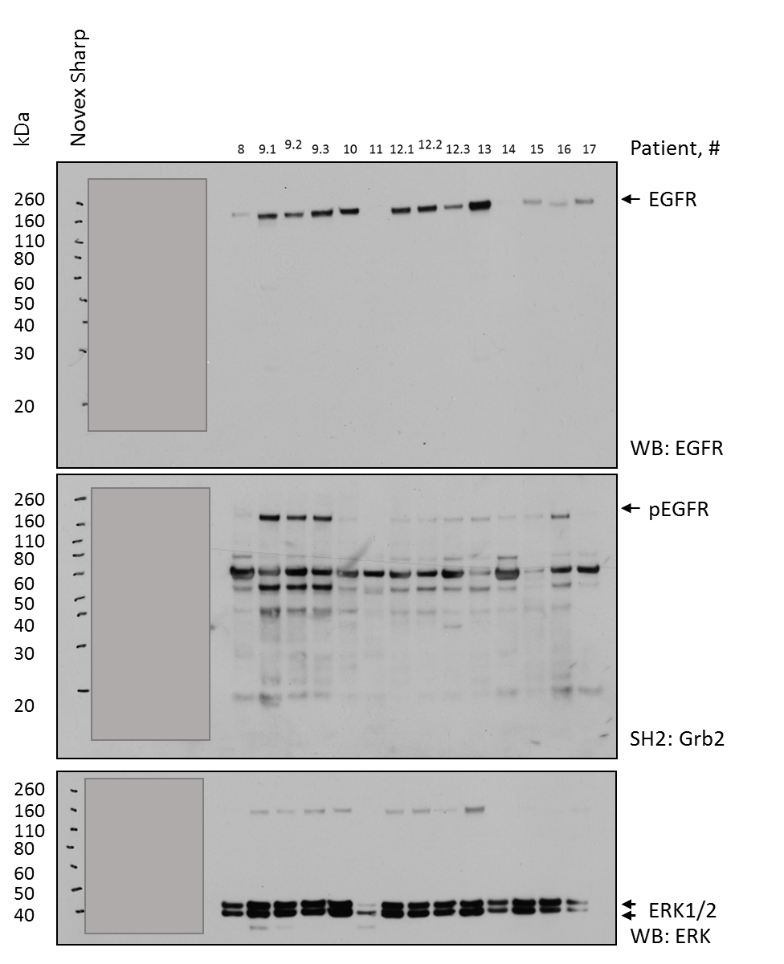


**Fig. S2 Uncropped Western and far-Western (SH2 profiling) blots from Fig. 2A. A** Western blots depicted in Fig. 2. The membranes were cut after blotting. The upper part was used for EGFR or pEGFR detection using fluorescent antibodies and the lower for detection of actin as a loading control. **B**For far-Western blots (SH2: Grb2) chemiluminescence was used for detection. **For A&B**: SAS cells were used as a standard to enable comparison of the different blots. Normal fibroblasts F180 and F184 were used as normal cell control. Additionally lysates from HeLa and Jurkat cells were detected to include also cells from different tumor entities. Lysate from F180 is partly missing on the third blot as indicated.

A


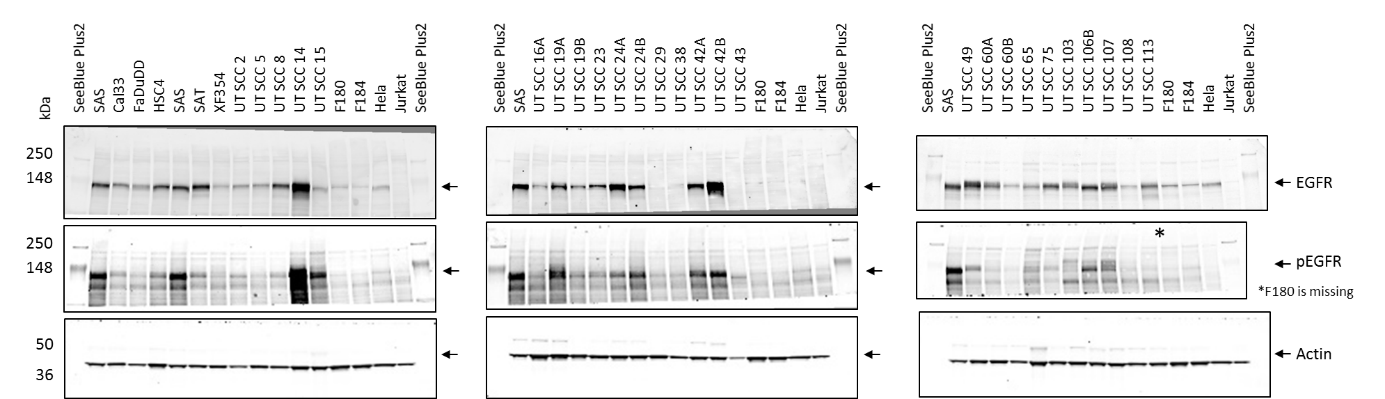


B


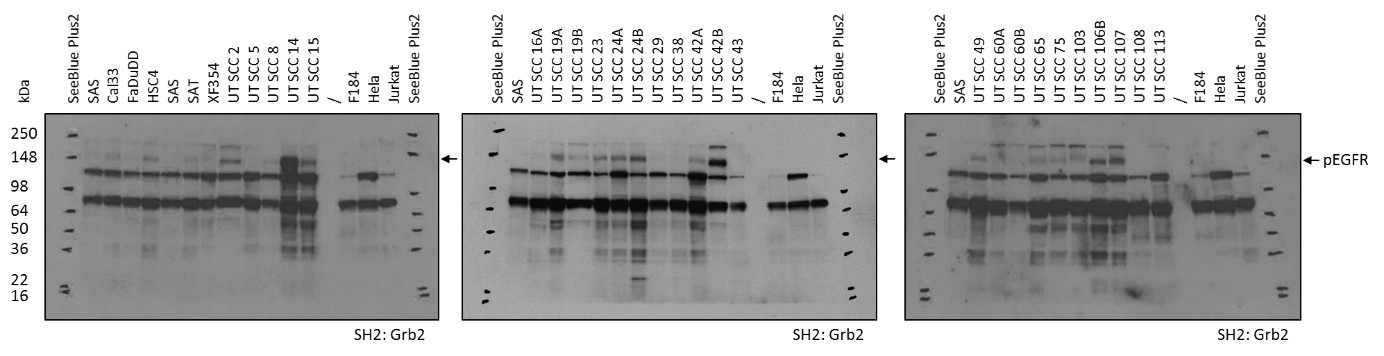


**Fig. S3 Uncropped Western and far-Western (SH2 profiling) blots from Fig. 3A.** The same lysates were used for blots shown in A and B. **A** Far-Western blot using different SH2 domains and Western blot using an ERK1/2 specific antibody. Signal detection was performed using chemiluminescence. The detection of ERK1/2 served as a loading control. **B** Western blot. The membrane was cut after blotting. The upper part was used for EGFR detection and the lower for detection of Actin as a loading control. Signal detection was performed using fluorescence labelled antibodies.

To improve clarity and conciseness of Fig. 3A intact blots from A and B were edited (brightness and contrast), cropped, rearranged and assembled.

A


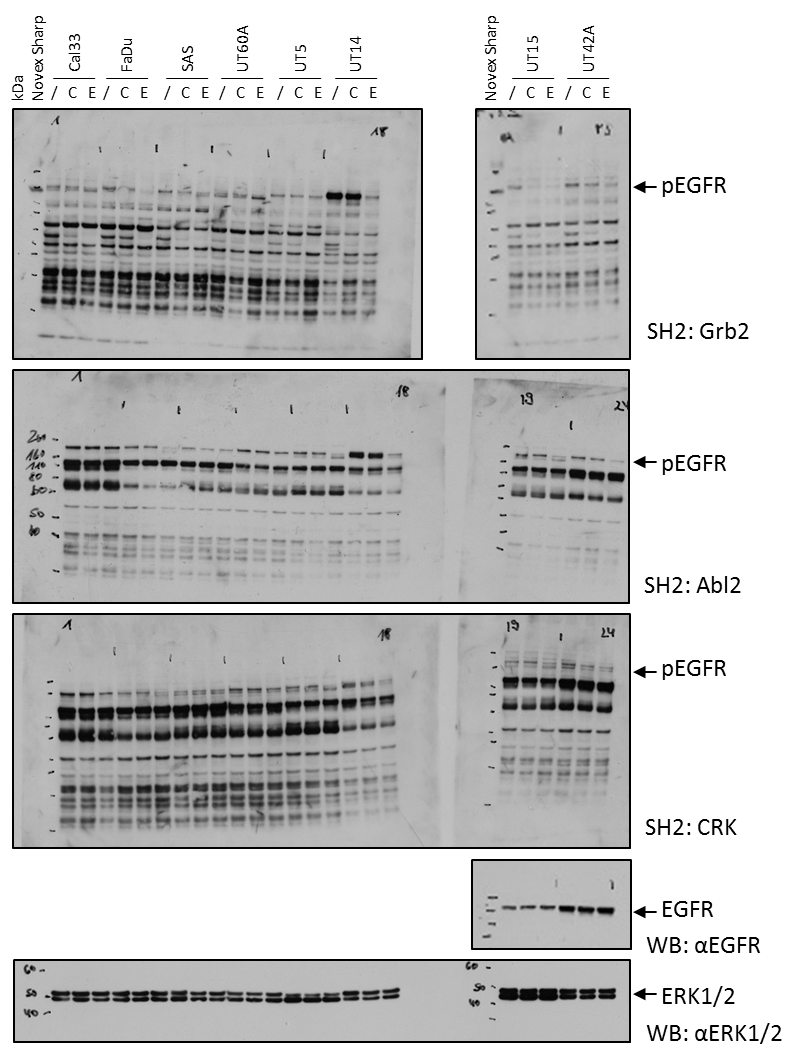


B


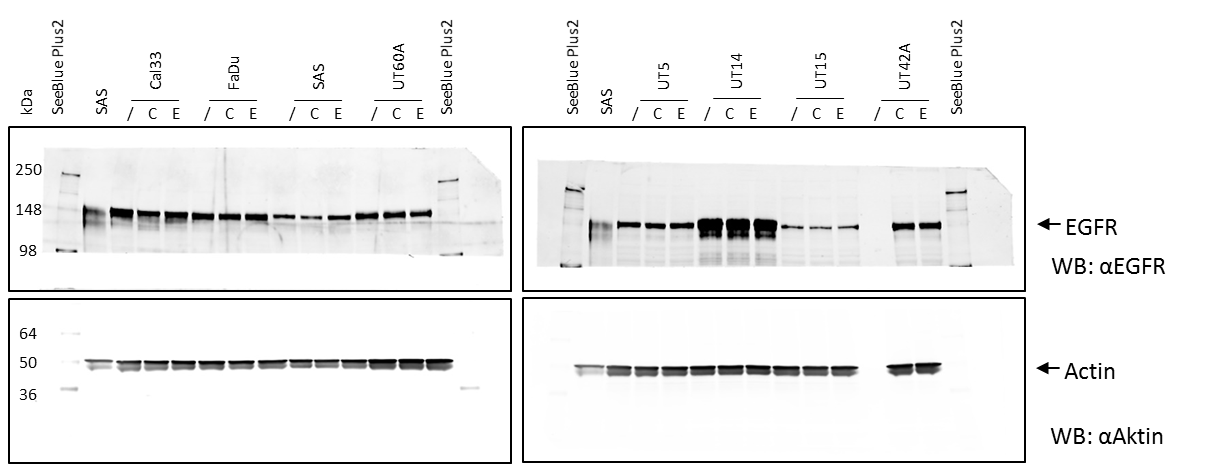

Supplement: Supplementary file 1 — Supplementary Dataset [file 41598_2019_49885_MOESM1_ESM.docx]
